# A Modular and Cost-Effective Droplet Microfluidic Device for Controlled Emulsion Production

**DOI:** 10.3390/polym16060765

**Published:** 2024-03-11

**Authors:** Hao Jiang, Zhaoyue Liu, Fengwei Tang, Yimin Cheng, Wei Tian, Woda Shi, Jia Ming Zhang, Yajun Zhang

**Affiliations:** 1 College of Mechanical and Electrical Engineering, Nanjing University of Aeronautics and Astronautics, Nanjing 210016, China; 2 Department of Cardiothoracic Surgery, Affiliated Hospital 6 of Nantong University, The Yancheng School of Clinical Medicine, Nanjing Medical University, Yancheng Third People’s Hospital, Yancheng 224000, China; 3 Yangtze River Delta Intelligent Manufacturing Innovation Center, Nanjing 210014, China

**Keywords:** droplet, modular, cost-effective, emulsion, microfluidics

## Abstract

The droplet microfluidic device has become a widely used tool in fields such as physics, chemistry, and biology, but its complexity has limited its widespread application. This report introduces a modular and cost-effective droplet microfluidic device for the controlled production of complex emulsions, including oil and aqueous single emulsions, and double emulsions with varying numbers of encapsulated droplets. The droplet sizes can be precisely controlled by easily replacing flat needles and adjusting the needle position within an axially accelerated co-flow field. This modular device not only allows for easy repair and maintenance in case of device clogging or damage but can also be readily expanded to produce complex emulsions. The low-cost and user-friendly nature of the device greatly facilitates the widespread adoption and utilization of droplet microfluidics.

## 1. Introduction

Droplet microfluidics is a cutting-edge technology that involves the controlled generation and manipulation of discrete droplets through immiscible multiphase flows within microchannels. This advanced technology has gained significant attention due to its remarkable advantages and has broad implications across various domains, including materials science [1], chemistry [2], and biology [3,4]. The attainment of precise control over droplets in microfluidic systems necessitates the use of microfluidic chips that contain microchannels, which play a crucial role in droplet generation and manipulation. Therefore, these chips are an indispensable prerequisite for research in droplet microfluidics [5].

The initial studies on microfluidic chips predominantly employed glass or silicon substrates in combination with standard photolithography [6]. However, the application of these substrates was hindered by their high cost and complex fabrication procedures [7]. Polymer materials have emerged as highly promising alternatives for fabricating microfluidic devices using mass-replication technologies, owing to their cost-effectiveness and ease of manipulation compared to glass-based substrates. Among the various manufacturing processes for polymer microfluidics, injection molding has been widely adopted [8]. Despite its suitability for large-scale production, injection molding necessitates a sophisticated and exacting process with stringent operational requirements [9]. Hot embossing is another commonly employed technique for fabricating polymer microfluidic chips [10,11,12]. However, one drawback of hot embossing is that it necessitates the production of molds beforehand. This process can be time consuming and often incurs substantial expenses [13]. In addition, micro-milling, as a microfabrication method for polymers, offers practical insights and strategies for rapid prototyping of microfluidic devices [14]. Nevertheless, it is important to note that micro-milling requires a significant amount of laboratory space and specialized technical training for operators. Additionally, the acquisition of large equipment for the milling process can result in high startup costs, limiting its widespread utilization in microfabrication [15].

The utilization of photosensitive resin has emerged as a highly promising polymer material in the field of vat photopolymerization. This breakthrough has resulted in rapid advancements and opened up exciting new possibilities for fabricating microfluidic chips. In addition, the introduction of stereolithography three-dimensional (3D) printing has played a significant role in reducing the overall fabrication time. By enabling the entire manufacturing process to be completed using a single, fully automated printing apparatus, this technology has revolutionized the efficiency and convenience of chip production [16]. Additionally, microfluidic chips fabricated from photosensitive resins exhibit favorable optical properties [17]. However, the current state of 3D printing technology presents significant challenges in microchannel fabrication, particularly within the domain of desktop photopolymerization printing techniques [18,19,20]. The exacting precision and diminutive dimensions of microchannels necessitate specialized industrial-grade and costly printing equipment, inevitably inflating the manufacturing expenses. Although desktop printers are available, their constrained accuracy and resolution render them poorly equipped to meet the manufacturing demands for microchannels, thus preventing them from producing droplet sizes as small as those generated by traditional microfluidic devices [21,22,23]. In addition, the current design of microfluidic chips is based on integration, which means that if any part of the chip becomes clogged or damaged, it becomes extremely difficult to replace or maintain. This issue often results in the entire microfluidic chip becoming unusable [24,25]. This inherent flaw not only leads to significant wastage but also imposes substantial economic burdens on users.

Recently, needle-based microfluidic devices have gained attention due to their simple fabrication manner. Lian et al. [26] used a needle-based microfluidic multiphase system to produce porous polydimethylsiloxane (PDMS) particles with different sizes and porosities; Li et al. [27] employed needles and Teflon tube to produce droplets that can be used for home-made microfluidic polymerase chain reactions (PCRs). Vijayan et al. [28] used different sized needles with commercial tubing to produce complex emulsions. These studies have demonstrated that it is feasible to use needles for droplet production.

In this study, we further exploit the potentials of needle-based microfluidic systems for complex droplet production. We propose a cost-effective and modular microfluidic chip for droplet generation. The key component of this device is a readily available and cost-effective flat needle with different diameters and a converging microchannel, which establishes an axially accelerated co-flow field structure. The generated droplet sizes can be adjusted by replacing flat needles with different diameters and further fine-tuning them by changing the position of the needle within this accelerated co-flow field, without modifying the fluid parameters. This flexible design significantly expands the range of droplet sizes that can be generated. This modular design has the capability to expand for the controlled production of complex emulsions with varying numbers of encapsulated droplets. Additionally, this innovative design prevents the adhesion of inner-phase droplets to the channel walls, eliminating the need for surface treatment. As a result, it enables the generation of both W/O or O/W single emulsions in the same device. This approach simplifies the complexity of microfluidic chip fabrication and reduces manufacturing costs. Unlike monolithic microfluidic devices, any damage or blockage within our modular device can be swiftly resolved by disassembling the components and replacing them with corresponding parts. Moreover, the accessibility of these readily obtainable components and the user-friendly nature of the structure make it well-suited for individuals lacking prior experience in droplet microfluidics. This aspect strongly supports the advancement and widespread implementation of droplet microfluidics.

## 2. Materials and Methods

This study presents a pioneering method for droplet generation through the application of an innovative axial acceleration co-flow field. In order to fully harness the capabilities of desktop 3D printing technology, functional microchannels featuring an axial acceleration co-flow field are manufactured. This is accomplished by integrating commercially available needles with printed microchannel modules, yielding a droplet microfluidic device tailored for precise emulsion production.

### 2.1. Fabrication of Plug-and-Play Droplet Microfluidic Device

To streamline the intricacies of conventional microfluidic chip fabrication, we have implemented a modular 3D printing manufacturing approach. This method not only guarantees the simplicity of the assembly and operation of the emulsion droplet generator but also leads to a reduction in manufacturing costs. The needle module and chip module of the emulsion droplet generator are designed using Solidworks (Dassault Systemes S.A., Vélizy-Villacoublay, France) and are then manufactured with the Form 3+ printer (Formlabs, Inc., Somerville, MA, USA). The dispersed phase channel, through which the inner phase flows, is designed as a circular channel with a diameter of 0.5 mm. Similarly, the continuous phase channel, through which the outer phase flows, is also a circular channel with a diameter of 1.8 mm. The matching of the inner and outer of a single emulsion droplet generator is achieved using a PTFE tubing connector with an outer diameter of 1.58 mm and an inner diameter of 0.75 mm. To improve the sealing capability, the use of 3D-printed gaskets is also integrated in this system. The threaded design on its own is inadequate for achieving comprehensive sealing and separation of distinct liquid phases. Modular assembly is carried out according to the specific requirements for producing different emulsions, with each module ensuring a secure sealing effect through the coordinated interaction of threaded structures and gaskets. The fabrication process utilized the Stereolithography (SLA) technique, which presents several advantages for the production of microfluidic devices. SLA employs an ultra-violet laser to achieve the photopolymerization of resins, leading to exceptional feature resolution and surface smoothness of the microchannels. These attributes play a critical role in enabling droplet formation and transportation within the microfluidic system. Moreover, the utilization of transparent resins in SLA enables the production of components with exceptional clarity following suitable post-processing. This optical transparency is particularly advantageous for microfluidic applications that necessitate visual examination. Through the use of transparent resins, researchers can acquire valuable insights into droplet behavior, thereby facilitating a comprehensive understanding of the fundamental processes and refinement of the system for improved performance. The clear resin provided by the printer company contains modified acrylate oligomer and monomer, together with a photoinitiator, but their weight ratio is proprietary. This synergistic combination of components contributes to the exceptional properties of the resin, rendering it highly suitable for microfluidic applications and observation within emulsion droplet generators.

### 2.2. General Equipment and Procedure

We employed Deionized (DI) water and water–glycerol mixtures (Sinopharm Chemical Reagent Co., Ltd., Shanghai, China) with different viscosities as the aqueous phases and silicon oils (Sigma-Aldrich, Singapore) of varying viscosities as the oil phases. The surfactant Tween 20 (Sinopharm Chemical Reagent Co., Ltd., Shanghai, China) was used in the aqueous phase, and the surfactant Span 80 (Wuxi Yatai United Chemical Co., Ltd., Wuxi, China) was used in the oil phase since this fluid is well soluble with silicone oil, which is used in our experiments as the oil phase. Both were added as volume fractions. Furthermore, green dyes were incorporated into the aqueous phase to facilitate improved visualization. The introduction of fluids into the chip at different flow rates was facilitated by the use of syringe pumps (LSP01-2A, Halma, Amersham, UK), with flow sensors from the same manufacturer employed to accurately measure the fluid flow rates. Specifically, in accordance with the liquid selection criteria outlined in Table 1 for the droplet generation experiment, the necessary liquids for creating oil and aqueous droplets were prepared. The experiment utilized a 3D-printed microfluidic chip and syringe needle assembly, with the components interconnected via fittings to establish the fluidic pathways for droplet generation. The syringe was affixed to a syringe pump, while the chip was secured on a displacement platform. The outer phase syringe pump was first activated to fill the microchannels with the outer phase solution. Following this, the inner phase syringe pump was initiated at a fixed flow rate of 5 μL/min. The outer phase flow rate was then adjusted to match the flow rate ratio, with a five-minute interval between each adjustment to ensure stable droplet generation. Once stability was achieved, the formation process of micro-droplets was captured using a high-speed camera (Rocketech Technology Corp., Ltd., Changsha, China). For our experimental setup, we acquired flat needles from Hongxin Electronic company. These flat needles are offered in different sizes. Here, we employed two distinct diameters: 34G (ID = 60 μm) and 30G (ID = 160 μm), providing us with the flexibility to choose the most suitable needle size for the production of droplets with different sizes. Upon completion of the experiment, the droplets were subsequently collected in a Petri dish for further analysis. Quantitative analysis of the droplet sizes was performed using image analysis software, ImageJ 1.52i. All experiments were carried out under a controlled room temperature of 22 °C to mitigate any potential environmental influences on the droplet generation process.

## 3. Results and Discussion

We undertake the design, fabrication, and assembly of plug-and-play droplet generators for the controlled production of single and double emulsions. In this work, we will offer a comprehensive overview of the specific structure of the droplet generator and the experimental process for droplet generation. Subsequent sections will delve into further details on these aspects.

### 3.1. Single Emulsion Generation

**Figure 1:**
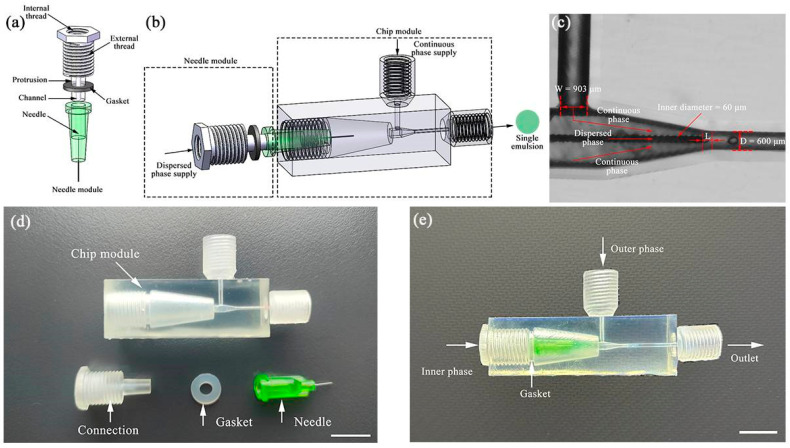
A modular microfluidic device for creating single emulsions. (**a**) Schematic of the needle module. (**b**) Schematic of the assembled device; (**c**) high-speed video frames of droplet formation: W represents the channel size of the continuous phase supply; D represents the outlet channel size; L represents the distance between the flat needle tip and the entry to the outlet channel; (**d**) all components to be assembled for creating single emulsions; (**e**) an assembled modular microfluidic device. Both scale bars in (**d**,**e**) are 1 mm.

The single emulsion generator comprises two modules: the needle module and the chip module. Figure 1a schematically displays the components of the needle module, including a connection part and a needle part. The connection part has an internal thread that connects with a commercial fitting for liquid supply, as well as an external thread that connects with the chip module. On the other hand, the needle part has a conical protrusion that fits flat needles, with a microchannel for liquid entry inside the protrusion. Furthermore, a gasket here ensures a tight seal, and the needle module is assembled tightly with the chip module. Figure 1b schematically shows the chip module, which includes a continuous phase supply channel, an outlet channel, and a converging channel. The continuous phase supply channel is vertically positioned on one side of the central axis and intersects with the horizontal channel to allow for the formation of droplets. Figure 1c obtained from high-speed recording images illustrates the process of droplet formation. A prismatic channel gradually narrows, away from the continuous phase supply channel. This three-dimensional co-flow structure prevents the dispersed phase from adhering to the channel walls, enabling the generation of both water-in-oil (W/O) and oil-in-water (O/W) emulsion droplets in the same chip without surface treatment. Additionally, by adjusting the position of the flat needle tip inside the prismatic channel, the droplet size produced can be tuned because the shear force at different locations of the prismatic channel varies. The generated emulsion droplets are collected through the outlet channel. As depicted in Figure 1d, we display all the components to be assembled for the single emulsion generator, and Figure 1e shows the assembled one.

**Figure 2:**
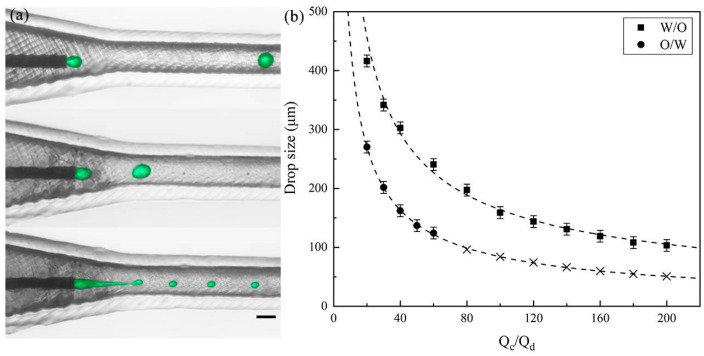
Generation of both W/O and O/W single emulsions. (**a**) High-speed video frames showing the droplet formation under different flow rate ratios. Color treatment applied to enhance observation. The scale bar is 400 μm. (**b**) Variation in sizes of both aqueous and oil droplets under different flow rate ratios.

We performed two sets of experiments using 60 μm sized needles to verify the practical efts. Firstly, we used 1 cSt silicon oil with 2% Span 80 as the dispersed phase and 50 cP water–glycerol mixtures with 2% Tween 20 as the continuous phase to generate oil droplets, fixing the dispersed phase flow rate (Q_d_) at 5 μL/min and gradually increasing the continuous phase flow rate (Q_c_). Figure 2a shows the formation of droplets with different flow rate ratios (Q_c_**/**Q_d_), while Figure 2b shows a decrease in droplet size from 300 μm to 100 μm as the flow rate ratio increases. Furthermore, we observed that by adjusting the flow rate, the size of the droplets varied within a limited range, as the flow rate cannot be infinitely high but is greatly influenced by the inner diameter of the needle. Beyond a flow rate ratio of 80, the shear force becomes too strong to produce oil droplets effectively. Subsequently, we used DI water with 2% Tween 20 as the dispersed phase and 50 cSt silicon oil with 2% Span 80 as the continuous phase to produce aqueous droplets. Following a similar procedure, the dispersed phase flow rate (Q_d_) was fixed at 5 μL/min, and the continuous phase flow rate (Q_c_) was gradually increased. Consequently, the droplet size decreased from 400 μm to 150 μm with increasing flow rate ratio (also seen in Supplementary Video S1), but aqueous droplets could still be produced until the flow rate ratio exceeded 200. Under the same flow rate ratio, the aqueous droplet size is larger than the size of the oil droplet. Notably, thanks to the clever design of the three-dimensional co-flow structure, we were able to generate both O/W and W/O emulsion droplets in the same microfluidic device without any surface treatment.

**Figure 3:**
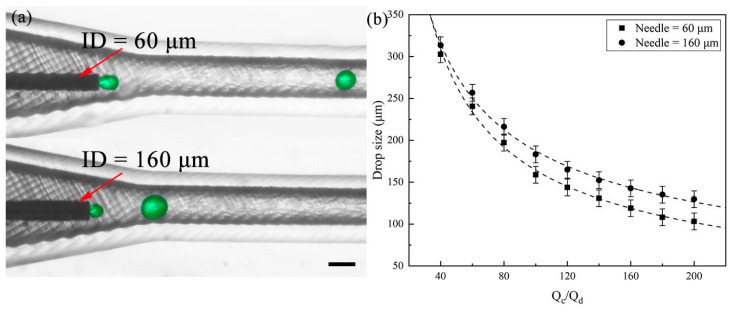
Generation of droplets via different needles. (**a**) High-speed video frames showing the droplet formation using flat needles of varying diameters. Color treatment applied to enhance observation. The scale bar is 400 μm. (**b**) Comparison of droplet sizes using different needles (34 G (60 μm) and 30 G (160 μm)) under different flow rate ratios.

We then explore the impact of different needle sizes on droplet formation in the subsequent experiments. Our plug-and-play microfluidic device allows for easy replacement of the flat needle, which serves as the core component for creating droplets. Here, we used DI water with 2% Tween 20 as the dispersed phase and 50 cSt silicone oil with 2% Span 80 as the continuous phase to produce aqueous droplets. Figure 3a illustrates droplet formation using two flat needle sizes: 34 G (60 μm) and 30 G (160 μm). By fixing the Q_d_ at 5 μL/min and increasing Q_c_, we produced different sizes of aqueous droplets, observing a reduction in droplet size as the flow rate of the continuous phase increased (also seen in Supplementary Video S2). With the same flow rate ratio, a smaller flat needle resulted in the creation of smaller droplets. This highlights the ability to precisely control droplet size by replacing different flat needle sizes without adjusting the fluid system and flow parameters. In addition, it is worth noting that both commercial and low-cost needles can be readily obtained and achieve feature sizes in the range of several tens of microns, allowing for the production of small droplet sizes. Creating such feature dimensions poses a formidable challenge when utilizing desktop-level equipment.

**Figure 4:**
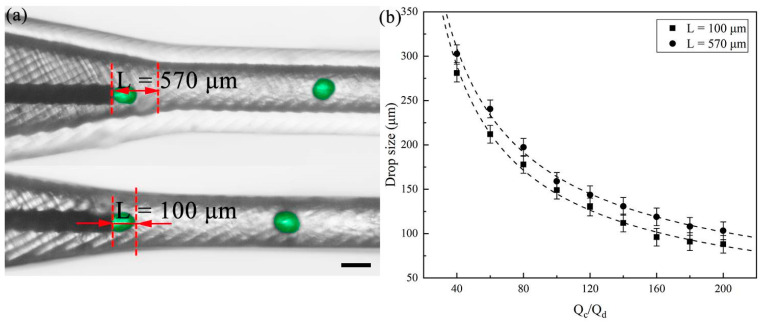
Generation of droplets at different needle positions. (**a**) High-speed video frames showing the droplet formation at different flat needle positions. Color treatment applied to enhance observation. The scale bar is 400 μm. (**b**) Comparison of droplet sizes at different needle positions under different flow rate ratios.

In addition, we investigated the effect of the position of the needle in the converging channel on the generation of droplets. The axially symmetric converging channel creates an accelerated co-flow field, leading to an increase in shear force as the fluid velocity rises due to the channel’s convergence. A 60 μm flat needle for droplet formation was employed here. We used DI water with 2% Tween 20 as the dispersed phase and 50 cSt silicone oil with 2% Span 80 as the continuous phase to produce aqueous droplets. Following a similar experimental procedure, we fixed the Q_d_ at 5 μL/min and gradually increased the Q_c_, resulting in a reduction in aqueous droplet size with an increase in the flow rate of the continuous phase. Figure 4a illustrates droplet formation with two typical flat needle positions, represented by the distance (L) between the flat needle tip and channel neck. The connection thread not only successfully connects both the needle and the chip module, but also enables control of the needle module movement. The needle position was precisely controlled by the thread pitch. We designed the thread with one pitch 500 μm, and we manually screwed it about half pitch each time. It resulted in the an adjustment of needle position ~250 μm. Different needle positions can be precisely adjusted (shown in Figure S1). The axially accelerated co-flow field we designed here introduces a shear force gradient along the flow direction. The precise adjustment of the needle positions in such field leads to controllable droplet sizes created due to their experience of different shear forces at different positions. As depicted in Figure 4b, when the flat needle is positioned in the narrow channel, the dispersed phase experiences an increased shear force, leading to a decrease in droplet size (also seen in Supplementary Video S3). We experimentally investigate the effect of adjusting the needle position on droplet sizes with respect to a large range of flow rate ratios. The droplet sizes are decreased more at the large flow ratio. The impact of needle position on droplet size is increased with increasing flow ratio due to the increase in shear force gradient.

**Figure 5:**
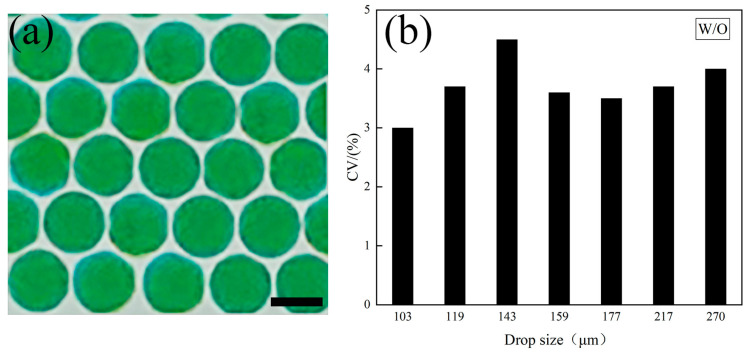
Droplet size analysis. (**a**) Optical micrograph of the monodisperse aqueous droplets. Green dyes are used here for enhancing observation. The scale bar is 250 μm. (**b**) The coefficient of variation (CV) for droplet size distribution. All CV values for different droplet sizes are lower than 5%.

Droplet formation is a crucial process that determines the quality and uniformity of droplets produced. In this study, the droplet formation is maintained in a dripping state, resulting in the production of highly uniform droplets. To analyze the droplets, we collected them in a Petri dish and analyzed their size distribution. In Figure 5a, we display the optical microscope image of the aqueous droplets, while Figure 5b presents the coefficient of variation (CV) value for the droplet size distribution. The CV is a statistical measure used to quantify the degree of variation in a data set. It is calculated as the ratio of the standard deviation (σ) to the mean diameter (d_av_) for the measured droplets [29]. To ensure the accuracy of their analysis, we conducted measurements and analysis of 100 droplets for each size, and the CV values for the droplets produced in our microfluidic device are consistently less than 5% for all sizes. This result suggests that the microfluidic device can produce droplets that are highly uniform in size and shape.

In conclusion, we create an accelerated co-flow field by utilizing the flat needle and converging channel. This innovative microfluidic device can stably produce both W/O and O/W emulsions without requiring any surface treatment. Additionally, the resulting droplet sizes are highly uniform and can be adjusted using various methods in our device. Adjusting the flow rate ratio can significantly alter the droplet size. Moreover, the plug-and-play design allows for easy flat needle replacement to achieve different droplet sizes. Furthermore, adjusting the needle position in the converging channel can further fine-tune the droplet sizes. This feature is particularly essential in situations where there are limited fluid parameter options, and it expands the applications of droplet microfluidics.

### 3.2. Double Emulsion Generation

**Figure 6:**
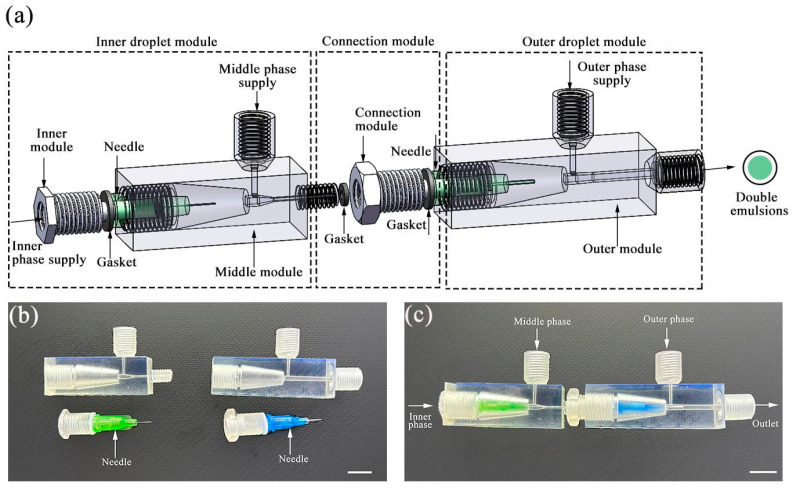
A modular microfluidic device for creating double emulsions. (**a**) Schematic of the modular microfluidic device including three modules: the inner droplet module, the outer droplet module, and the connection module. (**b**) All components to be assembled for production of double emulsions. (**c**) An assembled modular microfluidic device. Both scale bars in (**b**,**c**) are 1 mm.

Our design exhibits excellent scalability. We demonstrate that controllable double emulsions can be produced by adding an additional similar module. Specifically, the microfluidic device consists of three modules: the inner and outer droplet modules, along with a connection module. Figure 6a illustrates the schematic diagram of the double emulsion generation device. The connection module features both an internal thread connecting the inner droplet module and an external thread connecting the outer droplet module, with gaskets ensuring a secure seal. As depicted in Figure 6b, we display all the components required for producing double emulsions, while Figure 6c illustrates the assembled setup. Initially, the needle part was assembled for each droplet module, followed by the assembly of the two droplet modules. Simultaneously, the gasket is equally crucial as it ensures the sealing of each module, preventing any leakage during the emulsion generation process. It is worth noting that this consecutive module allows for the production of double emulsions with varying numbers of encapsulated droplets.

**Figure 7:**
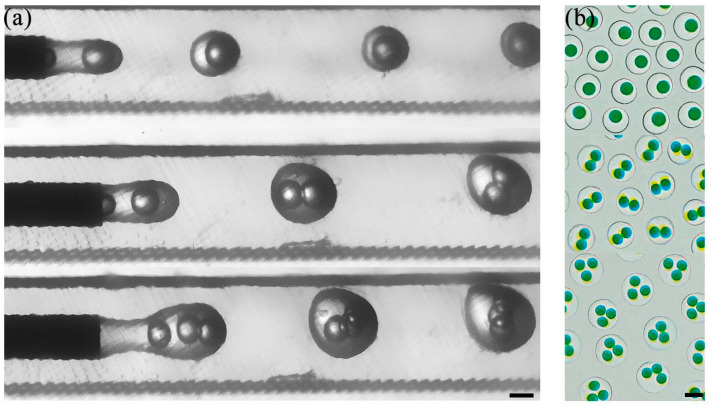
Generation of double emulsions with varying number of encapsulated droplets. (**a**) High-speed video frames showing the formation of double emulsion with 1, 2, and 3 encapsulated droplets. (**b**) Micrograph of the double emulsions. Both scale bars are 500 μm.

In the following experiments, based on the W/O/W liquid selection listed in Table 1, we used DI water (with 2% Tween 20) as the inner phase, 50 cSt silicone oil (with 2% Span 80) as the middle phase, and 220 cP water–glycerol mixture (with 2% Tween 20) as the outer phase to produce double emulsions. The flat needle size used here for inner droplet formation was 160 μm and for outer droplet formation was 410 μm. Both the oil phase and aqueous phase were introduced into the dispersed phase channels of the microfluidic chip via injection pumps. Firstly, the inner phase and intermediate phase converge at the needle outlet of the microfluidic chip, where the inner phase undergoes shear from the intermediate phase to form single droplets. Subsequently, the formed droplets pass through the connecting module and enter the dispersed phase channels of the next connected microfluidic chip, where the intermediate phase experiences shear from the outer phase to generate droplets, thus forming double emulsions. To achieve varying numbers of encapsulated droplets, careful adjustment of the flow rates for all phases is necessary. In this study, we kept the flow rate of the inner phase at 5 μL/min, while setting the flow rate of the middle phase at 16, 18, and 25 μL/min and the flow rate of the outer phase at 240, 200, 140 μL/min to create complex emulsions. The formed droplets enter the dispersed phase channel of the outer phase droplet generation chip through the connecting module. As depicted in Figure 7a, we successfully produced double emulsions with one, two, and three encapsulated droplets (also seen in Supplementary Video S4). In addition, Figure 7b presents microscope images of double emulsions with varying numbers of encapsulated droplets.

## 4. Conclusions

In this study, we have developed and manufactured a modular microfluidic device using a 3D-printed chip and low-cost flat needles. This device has demonstrated successful production of both W/O and O/W single emulsions without the need for any surface treatment. Moreover, it allows for the creation of controllable double emulsions with varying encapsulated droplets.

We highlight our modular approach that combines a 3D-printed converging channel with cost-effective flat needles to generate emulsion droplets. The utilization of low-cost flat needles allows for the production of small feature sizes that are typically challenging to fabricate using conventional desktop 3D printing equipment and can be easily replaced to produce different droplet sizes. In addition, we have introduced an axially accelerated co-flow structure, which enables further tuning of droplet formation by adjusting the flat needle position within this accelerated co-flow field, without needing to alter fluid parameters. Moreover, this modular design can be readily expanded to produce complex emulsions. An additional benefit of the modular device is its ability to facilitate effortless repairs and maintenance in the event of device clogging or damage, ensuring uninterrupted functionality.

These accessible components and user-friendly structures will benefit researchers without a background in droplet microfluidic technology, thereby facilitating the widespread adoption and utilization of droplet microfluidics.

## Figures and Tables

**Table 1:** Liquid selection for emulsion generation.

Phase	W/O	O/W	W/O/W
Inner	DI water	Silicone oil 1 cSt	DI water
Middle	—	—	Silicone oil 50 cSt
Outer	Silicone oil 50 cSt	DI water/Glycerin 50 cP	DI water/Glycerin 220 cP

## Data Availability

Data are contained within the article.

## Supplementary Materials

The following supporting information can be downloaded at: https://www.mdpi.com/article/10.3390/polym16060765/s1. Video S1: Generation of single emulsions under different flow rate ratios; Video S2: Generation of single emulsions with different needles; Video S3: Generation of single emulsions at different needle positions; Video S4: Generation of double emulsions with varying number of encapsulated droplets; Figure S1: Adjustment of needle positions in our designed microchannel.

## References

[1] Ho D.H., Song R., Sun Q., Park W.H., Kim S.Y., Pang C., Kim D.H., Kim S.Y., Lee J., Cho J.H. (2017). Crack-Enhanced Microfluidic Stretchable E-Skin Sensor. ACS Appl. Mater. Interfaces.

[2] Ayuso J.M., Virumbrales-Muñoz M., Lang J.M., Beebe D.J. (2022). A role for microfluidic systems in precision medicine. Nat. Commun..

[3] He F., Wang W., He X.H., Yang X.L., Li M., Xie R., Ju X.J., Liu Z., Chu L.Y. (2016). Controllable Multicompartmental Capsules with Distinct Cores and Shells for Synergistic Release. ACS Appl. Mater. Interfaces.

[4] Nunes J.K., Tsai S.S., Wan J., Stone H.A. (2013). Dripping and jetting in microfluidic multiphase flows applied to particle and fiber synthesis. J. Phys. D Appl. Phys..

[5] Shang L., Cheng Y., Zhao Y. (2017). Emerging Droplet Microfluidics. Chem. Rev..

[6] Kricka L.J. (2000). Microchips, Microarrays, Biochips and Nanochips—Personal Laboratories for the 21st Century. EJIFCC.

[7] Becker H., Gärtner C. (2000). Polymer microfabrication methods for microfluidic analytical applications. Electrophoresis.

[8] Dang F., Shinohara S., Tabata O., Yamaoka Y., Kurokawa M., Shinohara Y., Ishikawa M., Baba Y. (2005). Replica multichannel polymer chips with a network of sacrificial channels sealed by adhesive printing method. Lab Chip.

[9] Becker H., Gärtner C. (2008). Polymer microfabrication technologies for microfluidic systems. Anal. Bioanal. Chem..

[10] Lee U.N., Su X., Guckenberger D.J., Dostie A.M., Zhang T., Berthier E., Theberge A.B. (2018). Fundamentals of rapid injection molding for microfluidic cell-based assays. Lab Chip.

[11] Attia U.M., Marson S., Alcock J.R. (2009). Micro-injection moulding of polymer microfluidic devices. Microfluid. Nanofluid..

[12] Roy S., Yue C.Y., Venkatraman S.S., Ma L.L. (2013). Fabrication of smart COC chips: Advantages of N-vinylpyrrolidone (NVP) monomer over other hydrophilic monomers. Sens. Actuators B Chem..

[13] Shakeri A., Khan S., Jarad N.A., Didar T.F. (2022). The Fabrication and Bonding of Thermoplastic Microfluidics: A Review. Materials.

[14] Guckenberger D.J., Groot T.E., Wan A.M., Beebe D.J., Young E.W. (2015). Micromilling: A method for ultra-rapid prototyping of plastic microfluidic devices. Lab Chip.

[15] Aurich J.C., Reichenbach I.G., Schüler G. (2012). Manufacture and application of ultra-small micro end mills. CIRP Ann..

[16] Zhang J.M., Li E.Q., Aguirre-Pablo A.A., Thoroddsen S.T. (2016). A simple and low-cost fully 3D-printed non-planar emulsion generator. RSC Adv..

[17] Xu Y., Qi F., Mao H., Li S., Zhu Y., Gong J., Wang L., Malmstadt N., Chen Y. (2022). In-situ transfer vat photopolymerization for transparent microfluidic device fabrication. Nat. Commun..

[18] Zhang J.M., Ji Q., Duan H. (2019). Three-Dimensional Printed Devices in Droplet Microfluidics. Micromachines.

[19] Morimoto Y., Kiyosawa M., Takeuchi S. (2018). Three-dimensional printed microfluidic modules for design changeable coaxial microfluidic devices. Sens. Actuators B Chem..

[20] Li F., Macdonald N.P., Guijt R.M., Breadmore M.C. (2018). Increasing the functionalities of 3D printed microchemical devices by single material, multimaterial, and print-pause-print 3D printing. Lab Chip.

[21] Anna S.L. (2016). Droplets and Bubbles in Microfluidic Devices. Annu. Rev. Fluid Mech..

[22] Warr C.A., Hinnen H.S., Avery S., Cate R.J., Nordin G.P., Pitt W.G. (2021). 3D-Printed Microfluidic Droplet Generator with Hydrophilic and Hydrophobic Polymers. Micromachines.

[23] Li E.Q., Zhang J.M., Thoroddsen S.T. (2013). Simple and inexpensive microfluidic devices for the generation of monodisperse multiple emulsions. J. Micromech. Microeng..

[24] Niculescu A.G., Chircov C., Bîrcă A.C., Grumezescu A.M. (2021). Fabrication and Applications of Microfluidic Devices: A Review. Int. J. Mol. Sci..

[25] Juang Y.J., Chiu Y.J. (2022). Fabrication of Polymer Microfluidics: An Overview. Polymers.

[26] Lian Z., Ren Y., He J., Chen G.Z., Koh K.S. (2018). Microfluidic fabrication of porous polydimethylsiloxane microparticles for the treatment of toluene-contaminated water. Microfluid. Nanofluid..

[27] Li Y., Jiang Y., Wang K., Wu W. (2018). Passive Micropump for Highly Stable, Long-Termed, and Large Volume of Droplet Generation/Transport Inside 3D Microchannels Capable of Surfactant-Free and Droplet-Based Thermocycled Reverse Transcription-Polymerase Chain Reactions Based on a Single Thermostatic Heater. Anal. Chem..

[28] Vijayan S., Hashimoto M. (2019). 3D printed fittings and fluidic modules for customizable droplet generators. RSC Adv..

[29] Nie A., Cai Z., Gao Z., Wang Z., Eaglesham A. (2019). Droplet characteristics in the multi-staged high speed disperser with single inlet. Chin. J. Chem. Eng..

