# Supplementary material for: A Modular and Cost-Effective Droplet Microfluidic Device for Controlled Emulsion Production
Source: Polymers (Basel). 2024 Mar 11;16(6):765. doi: 10.3390/polym16060765 (PMC10974562; doi:10.3390/polym16060765)
Supplement: Supplementary file 1 [file polymers-16-00765-s001.zip › polymers-2814764-supplementary.pdf]

Supplementary Materials

A Modular and Cost-Effective Droplet Microfluidic Device for Controlled Emulsion Production

Authors

Hao Jiang[1,#], Zhaoyue Liu[1,#], Fengwei Tang[1], Yimin Cheng[1], Wei Tian[1], Woda Shi[2], Jia Ming Zhang[1,3,*], Yajun Zhang[2,*]

Affiliations

1 Affiliation 1: College of Mechanical and Electrical Engineering, Nanjing University of Aeronautics and Astronautics, Nanjing 210016, China;

2 Affiliation 2: Department of Cardiothoracic Surgery, Affiliated Hospital 6 of Nantong University, The Yancheng School of Clinical Medicine of Nanjing Medical University, Yancheng Third People's Hospital, Yancheng, 224000;

3 Affiliation 3: Yangtze River Delta Intelligent Manufacturing Innovation Center, Nanjing 210014, China;

# Hao Jiang and Zhaoyue Liu contributed equally;

* Correspondence: Zhang JM: zhangjmedu@163.com, Zhang Y: ycsyzyj@126.com;

This file includes:

1. Supplementary Video-S1:

Generation of single emulsions under different flow rate ratios.

This video demonstrates the process of producing W/O emulsions using the same needle at a needle position of L=570 μm under flow ratios of Q_c/Q_d=40 and 100. The video is recorded at 100 fps and played at 25 fps.

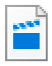

Video-1.mp4

2. Supplementary Video-S2:

Generation of single emulsions with different needles.

This video demonstrates the process of generating W/O emulsions using needles of different diameters (ID=60 μm and 160 μm) at the same flow ratio of Q_c/Q_d=80 and identical needle positions (L=570 μm). The video is recorded at 100 fps and played at 25 fps.

Video-2.mp4

3. Supplementary Video-S3:

Generation of single emulsions at different needle positions.

This video demonstrates the process of generating W/O emulsions using a 60 μm diameter needle at different needle positions (L=570 μm and 100 μm) at the same flow ratio of Q_c/Q_d=40. The video is recorded at 100 fps and played at 25 fps.

Video-3.mp4

4. Supplementary Video-S4:

Generation of double emulsions with varying number of encapsulated droplets.

This video demonstrates that double emulsions with 1-3 inner droplets are generated with different flow conditions. The flow conditions are (1) Q_I=5 μL/min, Q_M=16 μL/min, Q_O=240 μL/min, (2) Q_I=5 μL/min, Q_M=18 μl/min, Q_O=200 μl/min, (3) Q_I=5 μl/min, Q_M=25 μl/min, Q_O=140 μl/min, respectively. The video is recorded at 100 fps and played at 25 fps. (Q_I denotes the flow rate of the inner phase; Q_M denotes the flow rate of the middle phase; Q_O denotes the flow rate of the outer phase).

Video-4.mp4

5. **Supplementary figures**

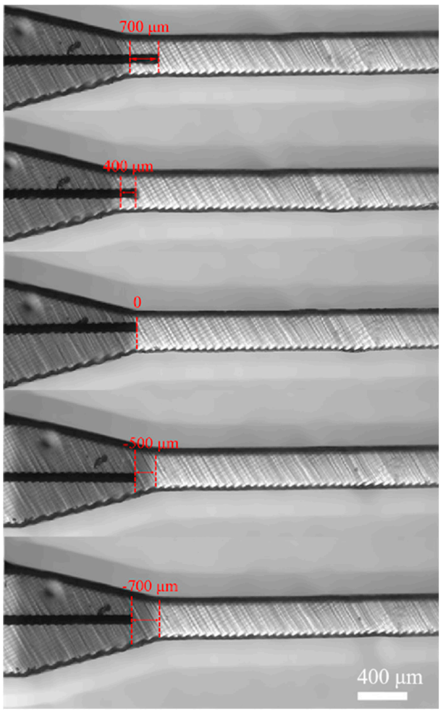

Figure S1 Adjustment of needle positions in our designed microchannel.
